# Parliament and the Profession

**Published:** 1916-08-19

**Authors:** 


### PARLIAMENT AND THE PROFESSION.

### Army Doctors in Egypt.

Temporarily commissioned R.A.M.C. officers serving in Egypt, who would on leaving to serve as doctors become subject to the provisions of the Military Service Act, have been retained beyond the termination of their year's contract. In making this statement, Mr. Forster added that their retention was necessary in order to provide adequately for medical requirements. It was hoped, however, that they might be able to return to this country at an early date when reliefs were available.

### Holidays for Examining Medical Officers.

Civil practitioners who were appointed examining medical officers, and have been so occupied under strain for a long period, are entitled to a holiday, said Mr. Forster, in reply to Mr. Touche. In answer to a further question, Mr. Forster stated that the maximum rate of pay for civil practitioners who are appointed examining medical officers is 40s. a day, the actual payments depending upon the number of recruits examined.

### Medical Equipment of the Army in India.

Asked by Sir Henry Craik what committees had been instituted within the last six years to inquire into the medical equipment of the Army in India, Mr. Chamberlain said that in 1912 a committee, of which Surgeon-General Sloggett was chairman, inquired into the system of field medical organisation and equipment of medical units, and its recommendations were approved by the Government of India and are now in force. The station hospital system for Indian troops was the subject of inquiry by another committee, whose recommendations were under discussion when the war broke out; as they related solely to peace arrangements, their further consideration has been postponed.

